# Vaginal Lactobacilli Induce Differentiation of Monocytic Precursors Toward Langerhans-like Cells: *in Vitro* Evidence

**DOI:** 10.3389/fimmu.2018.02437

**Published:** 2018-10-23

**Authors:** Jie Song, Fengchao Lang, Na Zhao, Yan Guo, Huatang Zhang

**Affiliations:** ^1^Yunnan Key Laboratory of Vaccine Research and Development on Severe Infectious Diseases, Institute of Medical Biology, Chinese Academy of Medical Science and Peking Union Medical College, Kunming, China; ^2^Chongqing Center for Biomedical Research and Equipment Development, Chongqing Academy of Science and Technology, Chongqing, China; ^3^Key Laboratory of Animal Models and Human Disease Mechanisms, Kunming Institute of Zoology, Chinese Academy of Sciences, Kunming, China; ^4^Kunming College of Life Science, University of Chinese Academy of Sciences, Kunming, China

**Keywords:** lactobacilli, monocytic precursors, Langerhans-like cell, peptidoglycan, HIV prevention

## Abstract

Lactobacilli have immunomodulatory mechanisms that affect the host cell immune system, leading to inhibition of HIV-1 transmission. Thus, lactobacilli as mucosal delivery vehicles for developing HIV-1 vaccines have attracted interest in recent years. Herein, we investigated the immunomodulatory effects of six strains of *Lactobacillus* naturally isolated from vaginal samples, including *Lactobacillus crispatus* (*L. crispatus), L. fermentum, L. jensenii, L. gasseri, L. delbrueckii* and *L. johnsonii*, on differentiation of monocytic precursors. *L. crispatus, L. fermentum* and *L. delbrueckii* could drive human monocytic cell line THP-1 cells to differentiate into dendritic-like cells according to the morphology. Moreover, *L. crispatus* increased costimulatory molecules including CD40, CD80 and CD86, and Langerhans cell specific C-type lectin receptors CD207, while *L. fermentum* decreased these molecules in THP-1 cells. Furthermore, *L. crispatus* promoted the differentiation of THP-1 cells with specific markers, phagocytic features, cytokine production ability and reduced the expression of receptors for HIV-1 entry of Langerhans cells. However, in the presence of *L. fermentum*, THP-1 cells did not show the above alterations. Moreover, similar effects of *L. crispatus* and *L. fermentum* were observed in CD14^+^ monocytes. These data suggested that *L. crispatus* facilitates the differentiation of monocytic precursors toward Langerhans-like cells *in vitro*. We further identified the cell wall components of *Lactobacillus* and found that peptidoglycans (PGNs), rather than bacteriocins, S-layer protein and lipoteichoic acid, were key contributors to the induction of CD207 expression. However, PGNs originating from *Bacillus subtilis, E. coli* JM109 and *E. coli* DH5α did not elevate CD207 expression, indicating that only PGN derived from *Lactobacillus* could enhance CD207 expression. Finally, the recognized receptors of *L. crispatus* (such as TLR2 and TLR6) and the upstream transcription factors (PU.1, TAL1, TIF1γ, and POLR2A) of CD207 were examined, and the expression of these molecules was enhanced in THP-1 cells following *L. crispatus* treatment. Thus, this study offers powerful evidence that vaginal lactobacilli modulate monocytic precursor differentiation into Langerhans-like cells probably via activating the TLR2/6-TFs-CD207 axis. These data provide clues for further investigation of the original occurrence, development and differentiation of Langerhans cells from monocytes.

## Introduction

Acquired immunodeficiency syndrome (AIDS) caused by human immunodeficiency virus-1 (HIV-1) remains one of the worst global health crises (1, 2). However, vaccines that prevent AIDS are not currently available. Thus, the development of a safe and effective HIV vaccine remains a major public health priority for preventing, controlling and ending the AIDS pandemic (3). Given that more than 90% of HIV-1 infections worldwide are transmitted at vaginal or rectal mucosal surfaces, strategies to develop HIV-1 vaccines have shifted focus from systemic immunity toward mucosal immunity (4). Furthermore, the microbiota of the lower female genital tract (FGT) is dominated by *Lactobacillus* species, which are considered an innate barrier to HIV-1 transmission, and depletion of vaginal lactobacilli is closely associated with the establishment of opportunistic infections and an increased risk of acquiring HIV-1 (5). The most frequently isolated lactic acid bacteria (LAB) species from healthy vagina include *Lactobacillus crispatus* (*L. crispatus*)*, L. gasseri, L. jensenii, L. fermentum, L. delbrueckii*, and *L. johnsonii* (6–8). These commensal bacteria not only secrete lactic acid to help maintain the low pH of the FGT environment but also produce hydrogen peroxide (H_2_O_2_), bacteriocins, and organic acids that have antimicrobial activity (9). Different strains of LAB were reported to have varying immunomodulatory properties and may finely regulate maturation, activation and functions of dendritic cells (DCs), monocytes, macrophages, and, to a minor extent, T cells (10). The development of DCs is affected by which microbes the precursors encounter through direct or indirect contact (11, 12). Different species of *Lactobacillus* possess the ability to regulate DC maturation, polarizing the subsequent T cell activity toward Th1, Th2, or Treg responses (13–19). In the immature stage, DCs express comparably low levels of CD40, CD80, CD86, and CD1a and do not express CD83. Immature DCs reside in peripheral tissues, continuously sampling the microenvironment, sensing the presence of pathogens, and releasing chemokines and cytokines to amplify the immune response (20, 21). Once immature DCs develop into mature DCs, they express high levels of CD40, CD80, CD86, and CD83. Mature DCs lose most of the antigen capture abilities but can stimulate T cell proliferation (22).

Langerhans cells (LCs) are a subpopulation of antigen-presenting DCs located in the epithelia, such as those of the skin, oral cavity, pharynx, esophagus, upper airways, urethra, and female reproductive tract, which are exposed to a wide variety of microbial pathogens (23, 24). LCs express high levels of CD207, CD205, E-cadherin, and CD1a and have Birbeck granules, which can degrade foreign antigens and restrict HIV-1 transmission at a low viral concentrations (25, 26). Based on these studies, we speculate that lactobacilli located at the mucosal surfaces of the FTG might have direct and/or indirect effects on the induction and differentiation of DCs.

In this study, we first used THP-1 cells, as well as CD14^+^ monocytes sorted from human peripheral blood mononuclear cells (PBMCs), for co-culture with 6 species of *Lactobacillus*, naturally isolated from swabs of the FTG and subsequently observed the immunomodulatory effects of *Lactobacillus* on the differentiation and maturation of THP-1 cells and CD14^+^ monocytes. This study will help elucidate the suppressive mechanism of lactobacilli on the spread of HIV and may have important implications for the development of mucosal HIV vaccines via lactobacilli as mucosal delivery vehicles.

## Materials and methods

### Isolation, culture and inactivation of bacterial strains

All the lactobacilli used in this study were originally isolated from vaginal swabs of healthy women using culture-based methods (27). The present study was approved by the Ethical Committee of the Second People's Hospital of Yunnan Province. All volunteers provided written consent and were informed of the purposes of the study. Six strains were identified as *L. crispatus, L. gasseri, L. jensenii, L. fermentum, L. delbrueckii* and *L. johnsonii* (detailed in the Results and Table S1). All strains were stored at −80°C in MRS media containing 30% glycerol (v/v) and plated on agarose containing MRS media (Hopebio, Qingdao, China) at 37°C for 96 h before individual colonies were used for routine cultures in liquid MRS medium (27).

For the indicated experiments, bacterial cells were exposed to ^60^Co radiation (10 KGy) for 10 h, and inactivation was assessed by inoculating MRS plates. *E. coli* JM109, *E. coli* DH5α, and *B. subtilis* purchased from Sigma-Aldrich (Gallen, Switzerland) were used as controls.

### Extraction of bacteriocins and cell wall components

Live and dead *L*. *crispatus* strains were pretreated with lysozyme (1 mg/ml, Sangon, Shanghai, China) for 1 h to degrade the β-1, 4 glucosidic bond between N-acetylmuramic acid and N-acetylglucosamine of the cell wall. Bacteriocins, S-layer protein (SLP), peptidoglycan (PGN) and lipoteichoic acid (LTA) were extracted from live and radiation-inactivated lactobacilli exactly as previously described (28–30). All extracts were assessed by sodium dodecyl sulfate polyacrylamide gel electrophoresis (SDS-PAGE) for their yield and purity and resuspended with phosphate-buffered saline (PBS) at 1 mg/ml. PGN was also extracted from *E. coli* JM109 and DH5α and used as controls.

### Isolation and culture of cells

Human monocytic leukemia THP-1 cells were purchased from Conservation Genetics CAS Kunming Cell Bank and were originally from the American Type Culture Collection (ATCC) (31). CD14^+^ monocytes and CD4+ T cells were sorted from PBMCs of healthy blood donors by magnetic separation with the Human Pan Monocyte Isolation Kit and Human CD4^+^ T Cell Isolation Kit (Miltenyi Biotec, Cologne, Germany) according to the manufacturer's instructions. All cell preparations were monitored by flow cytometry (BD FACSCalibur™, USA), and only those with purities greater than 95% were used for subsequent experiments. All cells were routinely cultivated in RPMI 1640 medium (Gibco, Waltham, USA) supplemented with 10% fetal bovine serum (FBS, Gibco, Waltham, USA) at 37°C in 5% CO_2_ in a humidified incubator with or without antibiotics as indicated.

### Co-culture and stimulation of cells with bacteria and extracted components

THP-1 cells or CD14^+^ monocytes were seeded in 6-well plates at a density of 2 × 10^5^ per well, and the selected lactobacilli were added at specified cell/bacteria ratios in antibiotic-free media for 4 h. Penicillin (100 U/ml) and streptomycin (100 U/ml) were then added to the co-culture system followed by incubation for the indicated times.

### Flow cytometry

Flow cytometry was routinely used to detect the expression of surface markers, including costimulatory molecules (CD40, CD80, CD83, CD1a, and HLA-DR), C-type lectin receptors (CD207, CD209, CD206, CD205, and CD303), LC-related markers (CD1a and CCR6), and HIV-1 entry receptors (CD4, CCR5, and CXCR4), Toll like receptor 1, 2, 4, and 6 (TLR1, 2, 4, and 6) and production of Th1- or Th2-associated cytokines (IFN-γ, TNF-α, IL-12, IL-4, and IL-10). Prospective antibodies conjugated with phycoerythrin (PE) and fluorescein isothiocyanate (FITC) were all purchased from BD Biosciences (Franklin, USA). The antibody-labeled cells were detected with a BD FACSCalibur™ flow cytometer (BD Biosciences, Franklin, USA) and analyzed using FlowJo7.6 software.

### Cytokine-induced differentiation and generation of DCs and LCs

DCs and LCs induced with cytokines were used as positive controls to monitor the experimental set up. Specifically, THP-1 cells and PBMC-derived CD14+ monocytes were cultured in 6-well plates with 100 ng/ml GM-CSF, 10 ng/ml IL-4 and 10 ng/ml TGF-β_1_ or with 100 ng/ml GM-CSF and 10 ng/ml IL-15 (all from PeproTech, Rocky Hill, USA), respectively, for 6 days. Induced cell samples were then monitored for expression of representative markers, including CD207, CD1a, and CCR6, by flow cytometry.

### Endocytosis assay

Following treatment with the selected lactobacilli, THP-1 cells (10^6^ cells/ml) were suspended in prewarmed serum-free RPMI 1640 medium, and FITC-conjugated dextran (Sigma-Aldrich, USA) was added at a final concentration of 1 mg/ml, followed by incubation for 1 h at 37°C. The cells were then washed three times with ice-cold PBS. Finally, samples in a volume of 300 μl of PBS were assayed by flow cytometry.

### Mixed lymphocyte reaction (MLR) and allogenic T-cell proliferation activity assay

MLR assays were set up using the THP-1 cells activated by lactobacilli and CD4^+^ T cells stained with CFSE (Crystal Field Stabilization Energies, CFSE). Specifically, 100 μl THP-1 cells (1 × 10^5^, 1 × 10^6^, and 1 × 10^7^) and 100 μl CD4^+^ T cells (1 × 10^5^) were mixed in a 6-well plate and co-cultured for 6 days; each assay was performed in triplicate. After centrifugation at 1,000 rpm for 2 min, cell pellets were resuspended in PBS. The MFI (mean fluorescence intensity) of total cells were detected by flow cytometry.

### Light and transmission electron microscopy (TEM) imaging

Changes in the morphology of the THP-1 cells following stimulation with lactobacilli were observed under a light microscope (Nikon, Tokyo, Japan). Selected lactobacilli and THP-1 cells, treated and untreated as indicated, were sequentially fixed with 2.5% glutaraldehyde and 1% osmium tetroxide (OsO_4_) in 0.1 M cacodylate buffer (pH 7.4). After dehydration with graded ethanol, cells were embedded in araldite (Sigma-Aldrich, Gallen, Switzerland) and sectioned. Ultrathin sections (4 μm) were counterstained with uranyl acetate and lead citrate and examined with a Leica electron microscope (Leica, Solms, Germany).

### Western blotting (WB) detection

Cell extracts containing 30 μg of proteins were fractionated on 8~15% SDS-PAGE gels by electrophoresis and electrophoretically transferred to polyvinylidene difluoride (PVDF) membranes. The membranes were incubated at 4°C overnight with primary antibodies, including SLP (1:1000, BIOSS, Shanghai, China), PU.1 (1:1000, Abcam, Cambridge, USA), TAL1 (1:1000, Abcam, Cambridge, USA), TIF1γ (1:1000, California, Santa Cruz, USA), POLR2A (1:1000, Abcam, Cambridge, USA) and β-actin (as a loading control, 1:1000, Abmart, Shanghai, China). Then, the blots were incubated with horseradish peroxidase-conjugated goat anti-mouse IgG (1:12000, Abmart, Shanghai, China) or goat anti-rabbit IgG (1:12000, Abmart, Shanghai, China). Bands were visualized with enhanced chemiluminescence reagents (Beyotime, Shanghai, China) and exposed with Kodak film for 1 min without light.

### Statistical analysis

All experiments were repeated at least three times, with representative results shown. The data are expressed as the mean ± standard deviation (SD). Statistical analysis was performed using SPSS software (Version 19, Italy). The differences between experimental groups were analyzed using ANOVA followed by Dunnett's multiple comparison tests with *P*-values less than 0.05 considered significant.

## Results

### Natural isolates of vaginal lactobacilli induced morphological changes similar to dendritic cells in THP-1 cells

**Figure 1:**
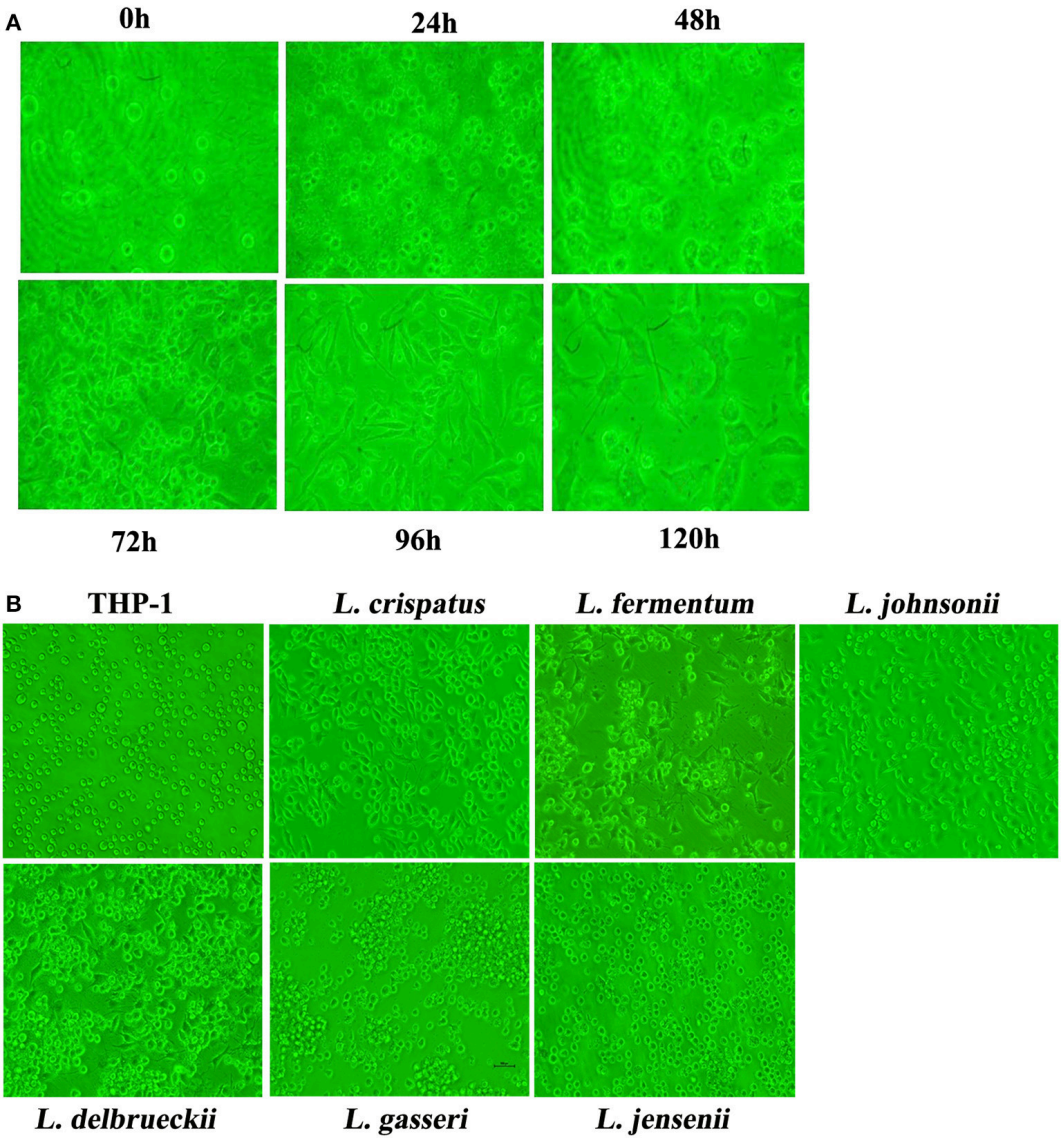
Morphology of THP-1 after stimulation with lactobacilli. **(A)** Morphology of THP-1 after stimulation with *L. crispatus*. THP-1 was incubated with live *L. crispatus* (ratio = 1:500) for the indicated times. Inverted microscopy image of cells in culture at a magnification of 200× is presented. **(B)** Morphology of THP-1 after stimulation with 6 species of *Lactobacillus*. THP-1 was incubated with different species of *Lactobacillus* (*L. crispatus, L. fermentum, L. jensenii, L. gasseri, L. delbrueckii*1, *and L. johnsonii*) (ratio = 1:500) for 96 h. Inverted microscopy image of cells in culture at a magnification of 200× is presented.

We successfully established continuous cultures of 11 isolates from 111 vaginal swabs from healthy females. In the initial attempt to investigate the effect of vaginal lactobacilli on the monocytic precursors of DCs, we arbitrarily chose *L. crispatus* to analyze CD80 expression by flow cytometry at different incubation ratios over time (Figure S1). As shown in Figure S1, under the incubation ratios 1:100, 1:300 and 1:500, CD80 expression gradually increased with time. Moreover, CD80 expression peaked following 96 h of co-culture at an incubation ratio of 1:500, which was subsequently chosen as the conditions for our investigations. The round appearance of THP-1 cells treated with *L. crispatus* at a concentration of 1:500 initially changed to a dendritic-like aspect at 72 h, which lasted to 96 h, but the THP-1 cells showed significant cell death at 120 h (Figure 1A). The morphological changes of THP-1 cells co-cultured with 5 other strains of lactobacilli are shown in Figure 1B. After co-culture for 96 h, most THP-1 cells treated with *L. crispatus, L. fermentum*, and *L. delbrueckii* showed adherence and exhibited a typical spherical shape with characteristic short hairy protrusions on their surface, suggesting that *L. crispatus, L. fermentum*, and *L. delbrueckii* promoted dendritic-like morphological alterations in the THP-1 cells.

### Lactobacilli stimulated monocytic precursors into immature DCs in THP-1 cells

**Figure 2:**
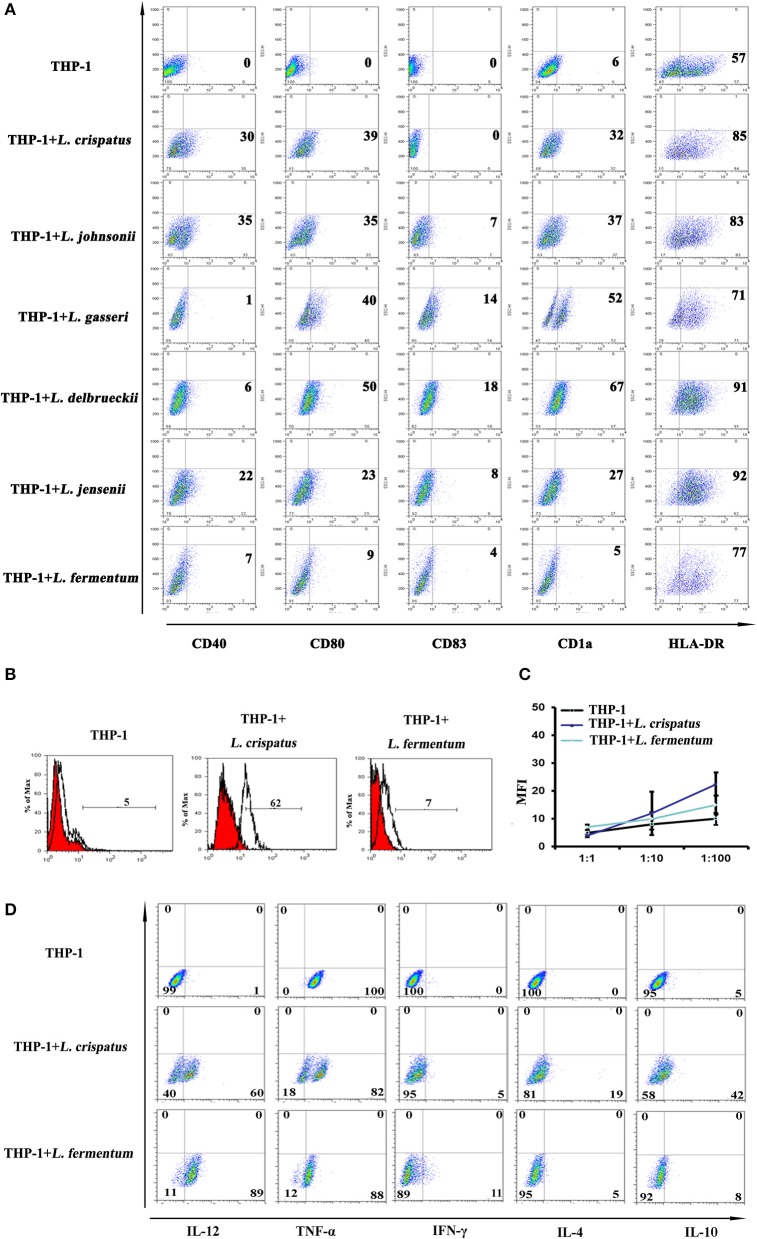
Dendritic cell characteristics on THP-1 after co-culture with lactobacilli. **(A)** Flow cytometric results showed CD40, CD80, CD1a, CD83, and HLA-DR expression on THP-1 cells stimulated with different species of *Lactobacillus* (*L. crispatus, L. fermentum, L. jensenii, L. gasseri, L. delbrueckii*1, *and L. johnsonii*) compared with unstimulated THP-1 cells. The percentages of positive cells in each analysis measured by MFI (mean fluorescence intensity) are presented. **(B)** Detection of THP-1 endocytic activity after stimulation with *L. crispatus* and *L. fermentum*. FITC-conjugated dextran positive cells were detected by flow cytometry. **(C)** Mixed lymphocyte reaction (MLR) analysis of THP-1 stimulated by *L. crispatus* and *L. fermentum*. 100 μl THP-1 cells (1 × 10^5^, 1 × 10^6^, and 1 × 10^7^) treated with *L. crispatus* and *L. fermentum* and 100 μl CD4^+^ T cells (1 × 10^5^) stained with CFSE were mixed in a 96-well plate and co-cultured for 6 day. The MFI were measured by flow cytometry and the results were recorded as the mean±SD from triplicate wells. **(D)** Intracellular cytokines of THP-1 after stimulation with *L. crispatus* and *L. fermentum*. THP-1 cells activated by *L. crispatus* and *L. fermentum* were harvested and stained with Th1- or Th2-associated cytokines (IFN-γ, TNF-α, IL-12, IL-4, and IL-10) kit.

CD40 expression was higher in THP-1 cells co-cultured with *L. crispatus* and *L. johnsonii* than THP-1 cells alone. CD80 and CD1a were markedly augmented in THP-1 cells co-cultured with 5 strains of *Lactobacillus*, except *L. fermentum*. There were no differences in CD83 expression in any group, while HLA-DR expression was relatively high in the *Lactobacillus*-treated groups compared to the untreated group (Figure 2A). Then, phagocytic capacity is assessed by FITC-dextran uptake. Immature DCs exhibit a high capacity for antigen uptake, whereas mature DCs lose their phagocytic ability (32). As noted in Figure 2B, treatment of THP-1 cells with *L. crispatus* significantly enhanced FITC-dextran uptake, but an increase in FITC-dextran uptake of *L. fermentum*-treated THP-1 cells was not observed. This finding indicates a critical role for *L. crispatus* in regulating the phagocytic capacity of THP-1 cells. To test the functional consequences of *Lactobacillus*-dependent differentiated THP-1, we assessed the ability of THP-1 to trigger T cell proliferation. There was no significant difference in the T cell ratio of THP-1 cells incubated with *L. crispatus* and *L. fermentum* compared to THP-1 cells alone (Figure 2C). These data imply that THP-1 cells incubated with *L. crispatus* and *L. fermentum* did not have an increased capacity to induce T lymphocyte proliferation. To further examine the effects of *L. crispatus* and *L. fermentum* treatment on cytokine production associated with Th1 and Th2 cell immune responses, we examined the effect of cytokine expression in THP-1 cells by flow cytometry. The data showed that Th1 cytokines, including IL-12 and TNF-α, were upregulated in *L. crispatus-* and *L. fermentum*-treated THP-1 cells compared with control cells. Moreover, compared to the THP-1 cells alone, THP-1 cells co-cultured with *L. crispatus* showed modest production of Th2 cytokines (such as IL-4 and IL-10), while THP-1 cells co-cultured with *L. fermentum* displayed no significant changes in these Th2 cytokines (Figure 2D). Overall, these results indicated a Th1-predominant cytokine phenotype in THP-1 cells co-cultured with *L. fermentum*, but THP-1 cells co-cultured with *L. crispatus* could simultaneously induce both Th1- and Th2-cytokine phenotypes. Altogether, these data suggested that lactobacilli, except *L. fermentum*, could facilitate THP-1 cells to differentiate into immature DCs.

### *Lactobacilli* regulated C-type lectin receptor expression in THP-1 cells

**Figure 3:**
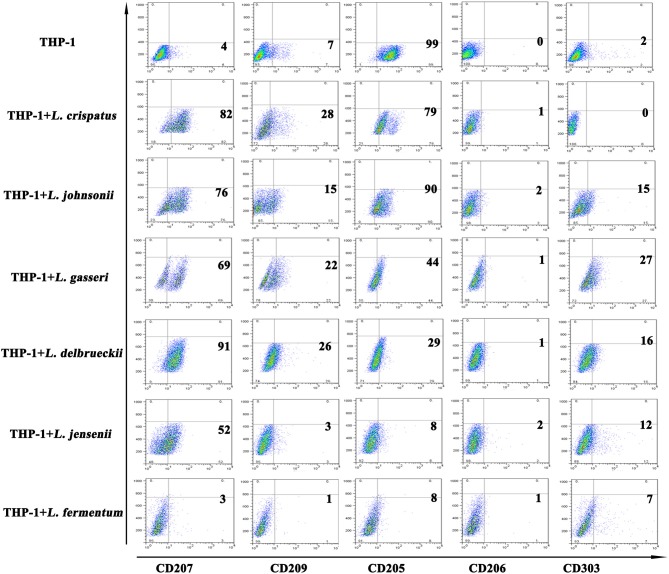
C-type lectin receptors on THP-1 after stimulation with lactobacilli. Flow cytometric results show CD207, CD209, CD205, CD206, and CD303 expression on THP-1 and monocytes stimulated with different species of *Lactobacillus* (*L. crispatus, L. fermentum, L. jensenii, L. gasseri, L. delbrueckii*, and *L. johnsonii*) compared to unstimulated THP-1 cells and monocytes.

C-type lectin receptors are differentially expressed depending on the subset of DCs (33, 34). Generally, Langerin (CD207) is specifically expressed on LCs and is the most notable marker of LCs. DC-SIGN (CD209) and MMR (CD206) are diversely distributed on the surface of dermal DCs. BDCA-2, also named CD303, is used to distinguish plasmacytoid DCs from other types of DCs. DEC-205 (CD205) is expressed on plasmacytoid DCs and LCs. We further detected C-type lectin receptors to better characterize the differentiated cells. We found that CD207 expression was significantly increased in THP-1 cells treated with 5 strains of *Lactobacillus*, except *L. fermentum*, compared with THP-1 cells alone; CD209 expression was elevated to a lesser extent in THP-1 cells treated with 4 strains of *Lactobacillus*, excluding *L. johnsonii* and *L. fermentum*; and CD303 expression was substantially upregulated in THP-1 cells treated with 5 strains of *Lactobacillus*, excluding *L. crispatus*. Additionally, there were no notable differences in CD206 expression between THP-1 cells alone and THP-1 cells stimulated with all 6 strains of *Lactobacillus*. CD205 expression in THP-1 cells stimulated with the 6 strains of *Lactobacillus* was significantly lower than that of THP-1 cells alone (Figure 3). These results indicated that different strains of *Lactobacillus* have different effects on the C-type lectin expression of THP-1 cells.

### *L. crispatus* facilitated LC surface marker expression in THP-1 cells

**Figure 4:**
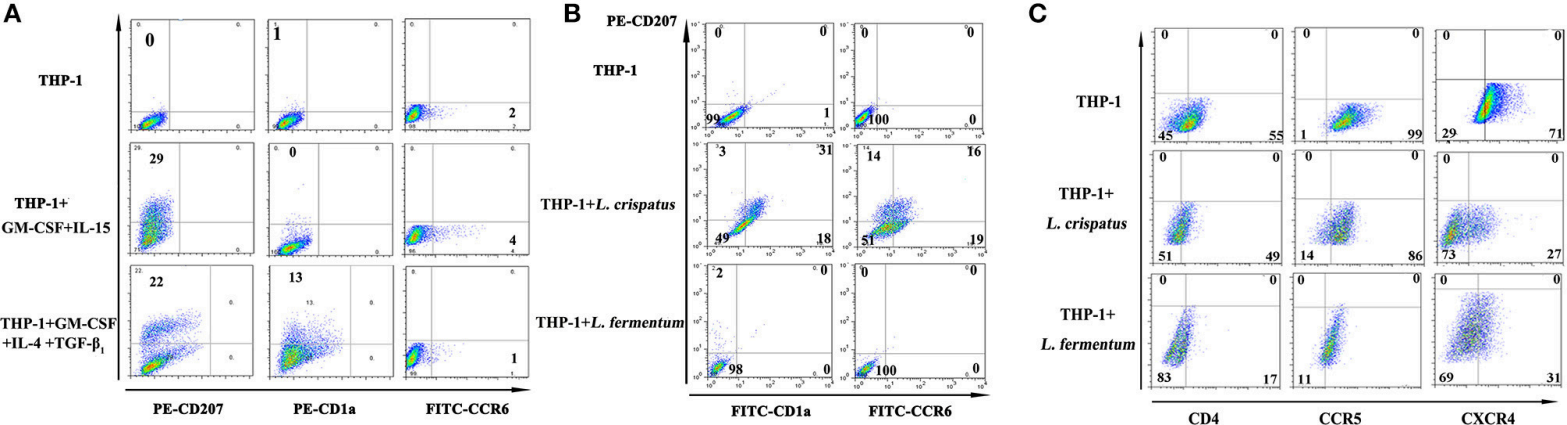
Langerhans cell markers and and HIV-1 receptors on THP-1 after stimulation with lactobacilli. **(A)** CD207, CD1a, and CCR6 expression on the surface of THP-1 stimulated with GM-CSF and IL-15 or GM-CSF, IL-4, and TGF-β_1_. **(B)** CD207, CD1a, and CCR6 expression on the surface of THP-1 stimulated with *L. crispatus* and *L. fermentum*. **(C)** HIV-1 receptors (CD4, CCR5, and CXCR4) expression on THP-1 after stimulation with *L. crispatus* and *L. fermentum*.

Based on the above results, which showed higher levels of costimulatory molecules (such as CD40, CD80, and CD1a) and C-type lectin receptors (such as CD207, CD209, and CD205) in THP-1 cells treated with *L. crispatus* but lower levels of these molecules in THP-1 cells treated with *L. fermentum*, we chose *L. crispatus* and *L. fermentum* to co-culture with THP-1 cells for the following experiments. As shown in Figure 4A, CD207 expression was dramatically increased in THP-1 cells responding to GM-CSF/IL-15 or GM-CSF/IL-4/TGF-β_1_, which could skew monocyte differentiation into LCs. CD1a was upregulated in GM-CSF/IL-4/TGF-β_1_-treated cells but not in GM-CSF/IL-15-treated cells. Low levels of CCR6 expression were found in GM-CSF/IL-4/TGF-β_1_- or GM-CSF/IL-15-treated cells. As illustrated in Figure 4B, CD207, CD1a, and CCR6 levels were markedly elevated in THP-1 cells following *L. crispatus* treatment, whereas CD207, CD1a, and CCR6 levels were not changed in THP-1 cells following *L. fermentum* treatment. These data suggested that *L. crispatus*, but not *L. fermentum*, induces differentiation of THP-1 cells into Langerhans-like cells. Additionally, TEM was used to observe Birbeck-like granules, which are specific organelles of LCs, in THP-1 cells co-cultured with *L. crispatus* and *L. fermentum*. However, Birbeck-like granules were not found in THP-1 cells co-cultured with *L. crispatus* and *L. fermentum* (Figure S2).

### *L. crispatus* suppressed the expression of HIV-1 entry receptors in THP-1 cells

CD4, CCR5, and CXCR4 were identified as the main cell receptors of HIV-1 (35), so the expression levels of these three molecules were evaluated in THP-1 cells stimulated with *L. crispatus* and *L. fermentum*. As summarized in Figure 4C, THP-1 cells stimulated with *L. crispatus* presented decreasing levels of CXCR4, along with a small decline in CD4 and CCR5, while THP-1 cells stimulated with *L. fermentum* showed strongly diminished CD4 and CXCR4 expression, with little reduction in CCR5. Thus, these data implied that *L. crispatus* and *L. fermentum* might induce alterations of HIV-1 entry receptors, which perhaps suppress HIV-1 entry.

### The expression of C-type lectins and HIV entry receptors in CD14^+^ monocytes incubated with *L. crispatus*

**Figure 5:**
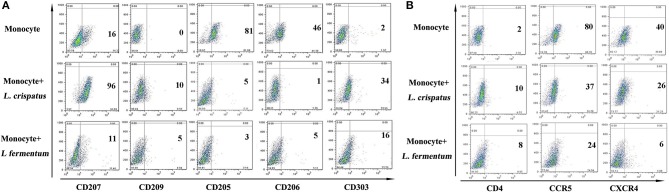
C-type lectins and HIV receptors in CD14^+^ monocytes after incubation with *L. crispatus* and *L. fermentum*. **(A)** Flow cytometry results showed CD207, CD209, CD205, CD206, and CD303 expression on CD14^+^ monocytes stimulated with *L. crispatus* and *L. fermentum* compared with unstimulated monocytes. **(B)** HIV-1 receptor (CD4, CCR5 and CXCR4) expression on CD14^+^ monocytes after stimulation with *L. crispatus* and *L. fermentum*.

Based on the above research, we also studied CD14^+^ monocytes for changes in C-type lectins and HIV entry receptors using flow cytometry. As shown in figure 5, CD14^+^ monocytes activated CD207, CD209, and CD303 expression in response to *L. crispatus*. However, CD205 and CD206 expression was notably inhibited. CD14^+^ monocytes treated with *L. fermentum* showed slight upregulation of CD303 expression and markedly suppressed CD205 expression. Additionally, CCR5 and CXCR4 were both significantly reduced in CD14^+^ monocytes following *L. crispatus* and *L. fermentum* treatments.

### Pgn of *L. crispatus*, rather than bacteriocins, SLP and LTA, was the key factor in promoting CD207 expression in THP-1 cells

**Figure 6:**
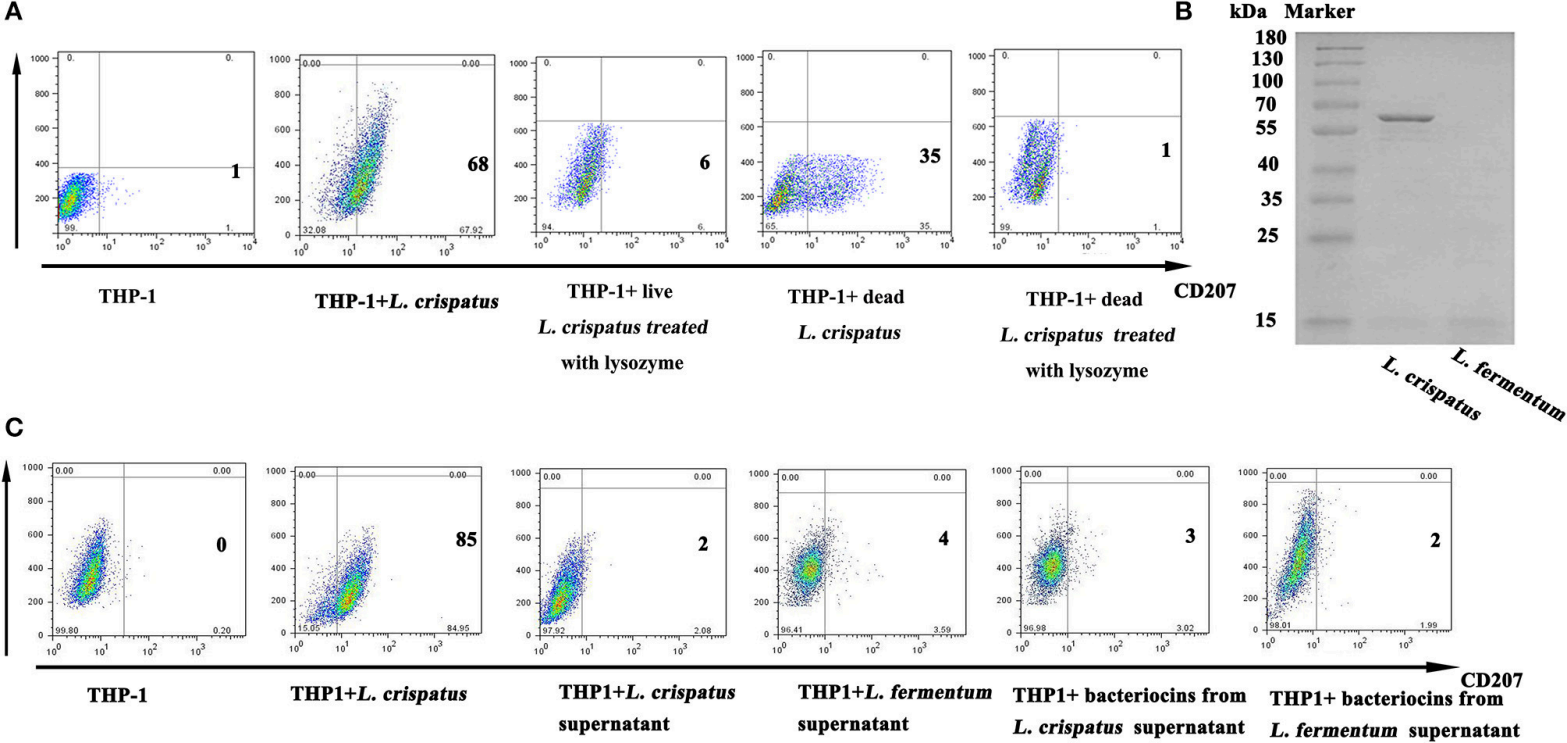
Expression of CD207 on THP-1 cells after stimulation with lactobacilli supernatant and cell components. **(A)** CD207 expression induced by *L. crispatus* inactived with radiation or lysozyme. **(B)** Purification of bacteriocins from *L. crispatus* and *L. fermentum*. Bacteriocins were isolated from the MRS medium and identified by SDS-PAGE. M indicates marker. **(C)** CD207 expression induced by bacteriocins from *L. crispatus* and *L. fermentum*. Cells were treated with supernatant of *L. crispatus* and *L. fermentum* (2×supernatant diluented with 2×RPMI 1640 at the ratio 1:1) and 1 mg/ml bacteriocins from *L. crispatus* and *L. fermentum*.

**Figure 7:**
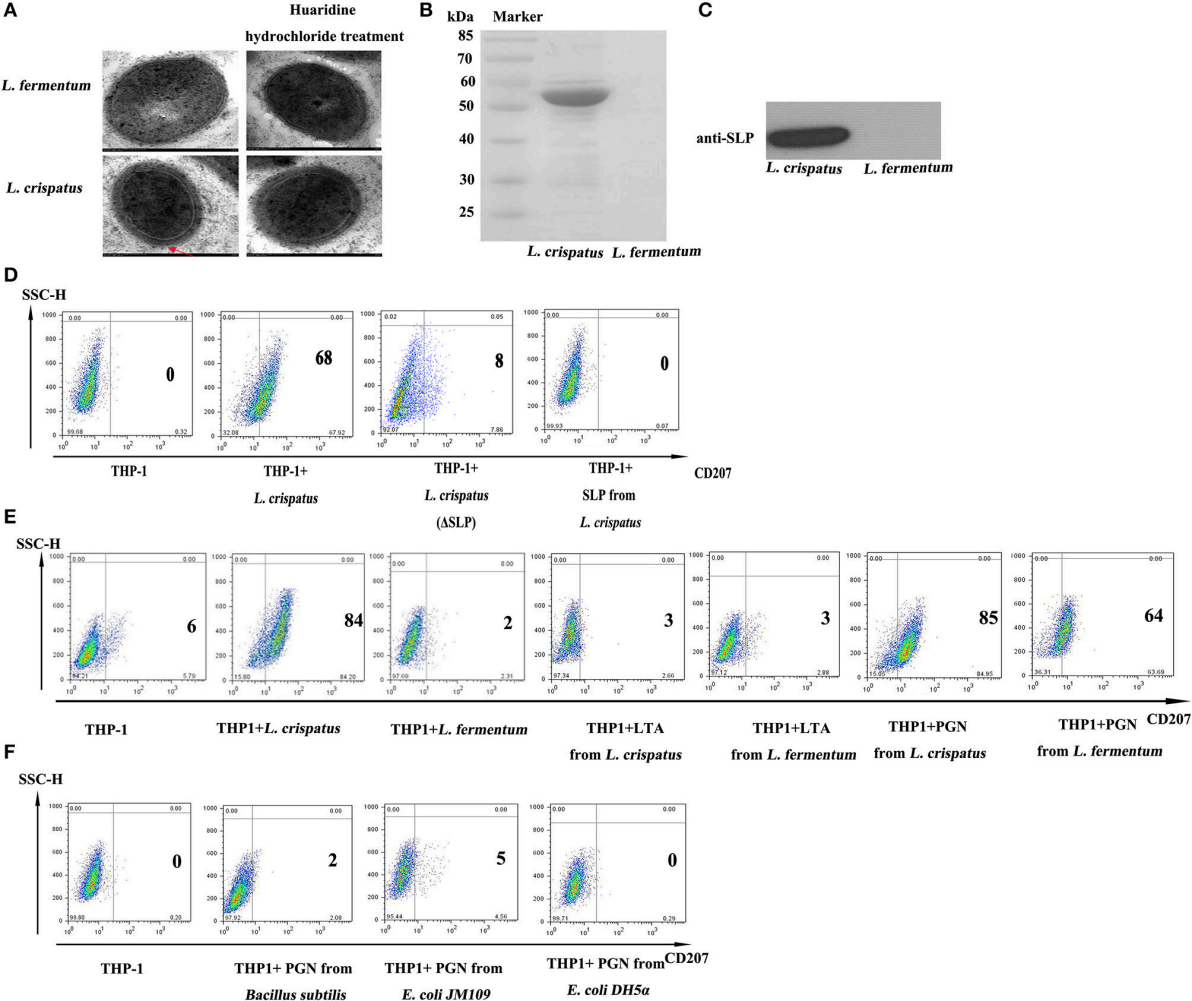
The function of SLP, f PGN and LTA in regulating the expression of CD207 on THP-1. **(A)** The structure of *L. crispatus* and *L. fermentum* were observed by transmission electron microscopy (TEM). *L. crispatus* and *L. fermentum* untreated and treated with huaridine hydrochloride were observed by TEM with high magnification. **(B)** Detection of SLPs from *L. crispatus* and *L. fermentum* by SDS-PAGE. **(C)** Identified SLPs from *L. crispatus* and *L. fermentum* by WB. The target protein is about 55 kDa. **(D)** CD207 expression induced by SLP from *L. crispatus*. Cells were treated with 1 mg/ml SLP from *L. crispatus* and *L. fermentum*. **(E)** CD207 expression induced by PGN and LTA from *L. crispatus* and *L. fermentum*. Cells were treated with 1 mg/ml PGN or LTA from *L. crispatus* and *L. fermentum*. **(F)** CD207 expression induced by PGNs from *Bacillus subtilis, E. coli* JM109 and *E. coli* DH5α. Cells were treated with 1 mg/ml PGN o from *Bacillus subtilis, E. coli* JM109, and *E. coli* DH5α.

As illustrated in Figure 6A, live (1:500) and dead *L. crispatus* (1:8000) both promoted CD207 expression in THP-1 cells, whereas live and dead *L. crispatus* treated with lysozyme did not enhance CD207 expression in THP-1 cells. This result suggests that the cell wall components may be responsible for CD207 expression. To further support the above conclusion, we concentrated bacteriocins from the supernatants of *L. crispatus* and *L. fermentum* and identified them by SDS-PAGE. Figure 6B shows that *L. crispatus* and *L. fermentum* can secrete several bacteriocins. Thus, the total supernatants from MRS medium and concentrated bacteriocins of *L. crispatus* and *L. fermentum* were incubated with THP-1 cells, and they did not activate CD207 expression in THP-1 cells (Figure 6C). We then investigated other cell wall components that might contribute to CD207 expression. First, SLPs of *L. crispatus* and *L. fermentum* were observed by TEM, which showed that *L. crispatus*, rather than *L. fermentum*, had an obvious SLP. After *L. crispatus* and *L. fermentum* were pretreated with guanidine hydrochloride, the SLP of *L. crispatus* disappeared (Figure 7A). Furthermore, the isolated SLP of *L. crispatus*, but not that of *L. fermentum*, was also detected by SDS-PAGE (Figure 7B) and WB (Figure 7C). Hence, analyses of the SLP and other elements, except the SLP (ΔSLP) of *L. crispatus*, which we used for co-culture with THP-1 cells, showed that the SLP and ΔSLP of *L. crispatus* cannot increase CD207 expression in THP-1 cells (Figure 7D). Subsequently, the extracted PGNs of *L. crispatus* and *L. fermentum* induced CD207 expression in THP-1 cells, while the extracted LTAs of *L. crispatus* and *L. fermentum* did not induce CD207 expression in THP-1 cells (Figure 7E). Moreover, to further identify whether the PGNs of all bacteria can induce CD207 expression in THP-1 cells, we chose two strains of Gram-negative bacteria, namely, *E. coli* DH5α and *E. coli* JM109, and one Gram-positive bacteria, *B. subtilis*. Their PGNs were extracted to co-culture with THP-1 cells. As shown in Figure 7F, the PGNs of *E. coli* DH5α, *E. coli* JM109, and *B. subtilis* could not elevate CD207 expression in THP-1 cells. This finding showed that PGN induces CD207 expression in a species-dependent manner.

### *L. crispatus* enhanced TLR2 and TLR6 expression in THP-1 cells in a concentration-dependent manner

**Figure 8:**
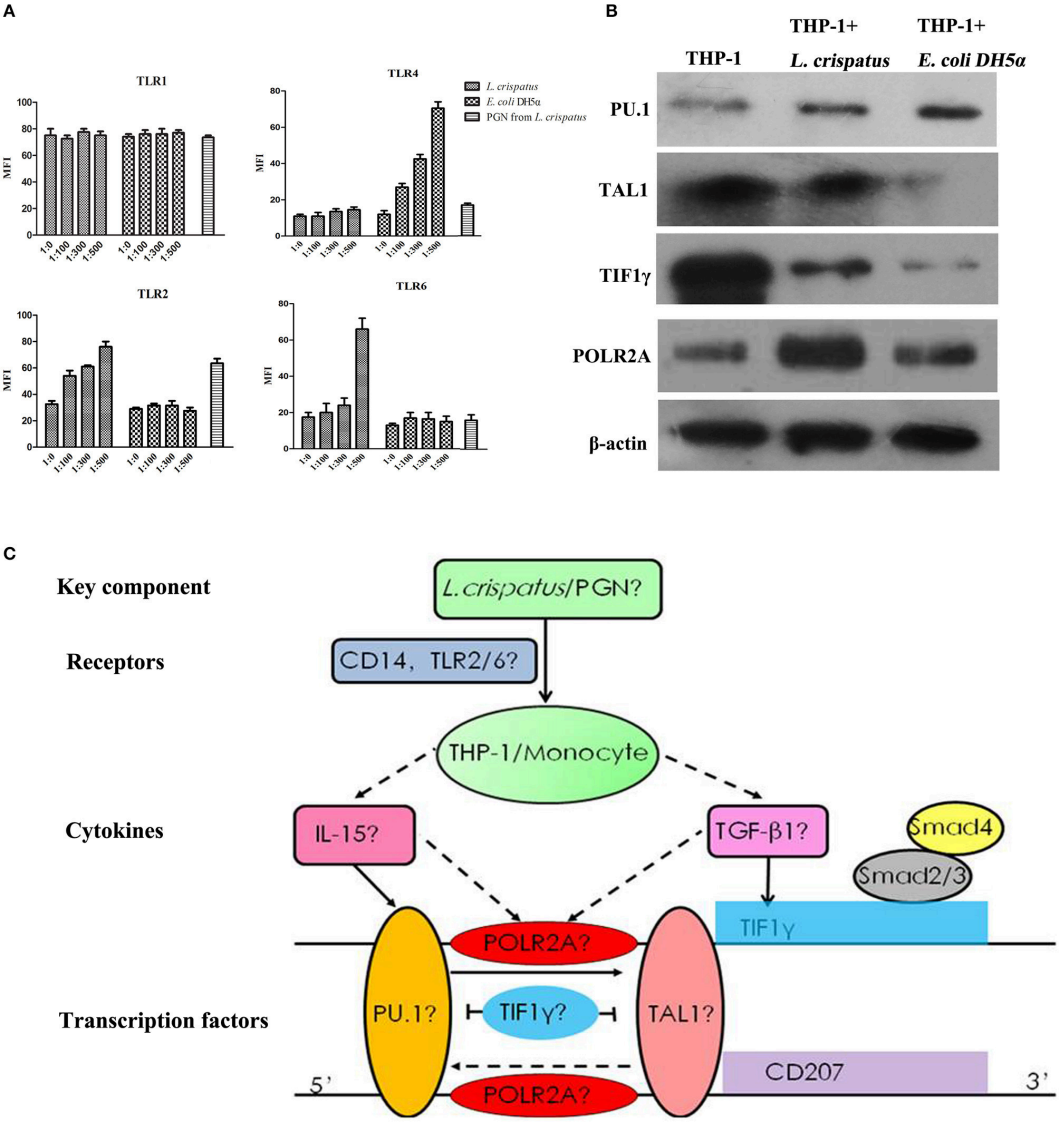
TLRs and transcription factors in THP-1 cells induced by *L. crispatus*. **(A)** *L. crispatus* enhanced TLR2 and TLR6 expression in THP-1 cells in a concentration-dependent manner. TLR1, 2, 4 and 6 were detected by flow cytometry after treated with *L. crispatus, E. coli* DH5α at the ratio of 1: 0, 1: 100, 1: 300, and 1: 500 or PGN from *L. crispatus* at 1 mg/ml. **(B)** Comparisons of the protein levels of PU.1, TAL1, TIF1γ, and POLR2A in THP-1 cells induced by *L. crispatus* and *E. coli* DH5α. **(C)** Roadmap for this study. *L. crispatus* might be recognized by TLR2 and TLR6 in THP-1 cells and then activate the related TFs of the GM-CSF with IL-15 or IL-4 and TGF-β_1_ signaling pathways, which can ultimately lead to CD207 expression.

To examine the effects of *L. crispatus* on TLR expression in THP-1 cells, we performed flow cytometry. As shown in Figure 8A, there were no significant differences in TLR1 and TLR4 expression among all *L. crispatus*-treated THP-1 groups. Nevertheless, the expression levels of TLR2 and TLR6 were gradually elevated in THP-1 cells following *L. crispatus* treatment with an increasing bacteria/cell ratio. Meanwhile, only the expression of TLR4 was increased after treated with *E. coli* DH5α. Hence, these data implied that *L. crispatus* treatment facilitated TLR2 and TLR6 expression in THP-1 cells in a concentration-dependent manner.

### Transcription factors (TFS), including PU.1, TAL1, TIF1γ and POLR2a, may be involved in the induction of CD207 in THP-1 cells stimulated with *L. crispatus*

We predicted the upstream TFs of CD207 using the University of California Santa Cruz (UCSC) genome browser (Human Dec. 2013 version) and discovered four key TFs, PU.1, TAL1, TIF1γ, and POLR2A, which may bind within 500 bp upstream and downstream of the CD207′ ORF (open reading frame, ORF) (36). And these four TFs are also components of the GM-CSF/IL-15 or GM-CSF/IL-4/TGF-β_1_ signaling pathways (37–40). WB analysis revealed that PU.1 and POLR2A protein levels were upregulated in THP-1 cells treated with *L. crispatus* and *E. coli* DH5α, whereas TAL1 and TIF1γ protein expression levels were downregulated in THP-1 cells treated with *L. crispatus* and *E. coli* DH5α compared to untreated THP-1 cells. Moreover, the upregulation degree of PU.1 in THP-1 cells treated with *L. crispatus* was clearly lower than that in THP-1 cells treated with *E. coli* DH5α, but the upregulation degree of POLR2A in THP-1 cells treated with *L. crispatus* was remarkably higher than that in THP-1 cells treated with *E. coli* DH5α. However, the downregulation of TAL1 and TIF1γ in THP-1 cells treated with *L. crispatus* was notably weaker than that in THP-1 cells treated with *E. coli* DH5α (Figure 8B).

## Discussion

In this study, we first observed the morphological changes in THP-1 cells treated with 6 stains of *Lactobacillus which* were previously isolated from vaginal swabs of healthy women. *L. crispatus, L. fermentum*, and *L. delbrueckii* could promote a dendritic-like morphological alteration of THP-1 cells. Subsequently, the surface molecules of DCs, including costimulatory molecules, antigen presentation molecules and C-type lectin receptors, were identified by flow cytometry. Our results showed that the costimulatory molecules (e.g., CD40 and CD80), antigen presentation molecules (e.g., HLA-DR) and C-type lectin receptors (e.g., CD207 and CD209) were significantly increased in THP-1 cells in response to *L. crispatus*, excluding DCs mature marker CD83. These phenotypes indicated that THP-1 cells stimulated by *L. crispatus* could differentiate into immature DCs. Additionally, the expression levels of CD207, CD1a, and CCR6, which are often used as LC markers, especially CD207, which is expressed uniquely in LCs, were notably elevated in THP-1 cells incubated with *L. crispatus*, implying that the immature DC subtype of THP-1 cells induced by *L. crispatus* might be Langerhans-like cells. LCs, as immature DCs, are also characterized by high endocytic activity and low T-cell activation potential. The phagocytic ability, unactivated mixed lymphocyte reaction and Th1/Th2 immune responses in this study revealed that THP-1 cells treated with *L. crispatus* had a strong phagocytic activity, could not efficiently induce naïve CD4^+^T cell proliferation and could promote Th1/Th2 immune responses, further implied that THP-1 cells stimulated by *L. crispatus* had features of immature DCs. Finally, we found that CD4, CCR5, and CXCR4 were remarkably downregulated, but Birbeck granules were not observed in THP-1 cells treated with *L. crispatus* (Figure S2).

The anti-HIV-1 function of LCs is not only due to the ability of CD207 to efficiently capture HIV-1 and rapidly internalize it into Birbeck granules, resulting in viral degradation, but also due to sequestration of HIV-1 to prevent interactions with its entry receptors, e.g., CD4, CCR5, and CXCR4. Thus, *downregulation of HIV-1 receptors* suggested that *L. crispatus* might indirectly prevent HIV-1 infection. However, since we did not perform the HIV-1 infection assay, these speculations need to be verified by inoculating *L. crispatus* in gnotobiotic animal models.

We further investigated some key components in *L. crispatus*, which contribute to the regulation of CD207, a specific marker of LCs. First, both live and dead *L. crispatus* could augment CD207 expression in THP-1 cells, indicating that the primary components responsible for CD207 expression were not present in excretions of *L. crispatus*. Moreover, the concentrated bacteriocins did not induce CD207 expression. Subsequently, lysozyme was used to degrade the cell wall of *L. crispatus*, and neither live *L. crispatus* nor dead *L. crispatus* improved CD207 expression, suggesting that the cell wall of *L. crispatus* was the key player. The cell wall of *L. crispatus* has a typical Gram-positive structure, which is made of a thick multilayered PGN sacculus decorated with proteins, LTAs and polysaccharides, and is surrounded in some species by an outer shell of proteins packed in a paracrystalline layer (S-layer) (41). Hence, we extracted different components of the cell wall of *L. crispatus* to screen for the key player, and since THP-1 cells treated with *L. fermentum* did not express CD207, we chose *L. fermentum* as a negative control. We found that SLP was present in *L. crispatus*, but not in *L. fermentum*, and SLP isolated from *L. crispatus* did not promote CD207 expression. Notably, in addition to the SLP of *L. crispatus*, the remaining components of the *L. crispatus* cell wall (that is, the ΔSLP group) unexpectedly did not increase CD207 expression. Although the detailed functions of SLP are still unclear, SLP as an adhesion protein mediating attachment or binding to different host surfaces has been described (42). Therefore, we surmised that SLP might help narrow the distance between PGN of *L. crispatus* and THP-1 cells to facilitate the recognition process of *L. crispatus* and THP-1.

LTAs did not elevate CD207 expression. Although LTAs have been reported to have a prominent role in host-lactobacilli interactions, especially adhesion, they were not a major component during the differentiation of THP-1 cells after *L. crispatus* treatment in this study (43). Finally, PGNs isolated from *L. crispatus* and *L. fermentum* enhanced CD207 expression, which indicated that PGN might be the key component involved in the differentiation of THP-1 cells. In addition, to further explore whether PGNs from all bacteria can upregulate CD207 expression in THP-1 cells, we selected 1 strain of Gram-positive bacterium and 2 strains of Gram-negative bacterium, namely, *B. subtilis, E. coli* JM109 and *E. coli* DH5α, and the data revealed that PGN extracted from the 3 strains of bacteria did not upregulate CD207 expression, suggesting that PGN induces CD207 expression in a species-dependent manner. Moreover, PGN isolated from *L. fermentum*, but not the whole *L. fermentum*, can trigger CD207 expression in THP-1 cells. This finding further indicates that PGN promoted the differentiation of THP-1 cells.

To explore the possible regulatory mechanism of PGN on CD207 expression in THP-1 cells, we assessed the PRRs, including TLR1, TLR2, TLR4, and TLR6, by flow cytometry in THP-1 cells following *L. crispatus* incubation. PGN is mainly recognized by TLR2, which is activated and exerts its function by the formation of heterodimers with TLR1 or TLR6 (44). In our results, TLR2 and TLR6 were upregulated in THP-1 cells following *L. crispatus* treatment. Thus, *L. crispatus* may might trigger the recognition process of THP-1 cells by TLR2 and TLR6. After activation of TLR2 and TLR6, the potential downstream pathway that mediated CD207 expression in THP-1 cells needs to be further investigated. We predicted the upstream TFs of CD207 using the UCSC genome browser and discovered four key TFs, including PU.1, TAL1, TIF1γ, and POLR2A, which may be involved in the regulation of CD207 expression. These four TFs are also components of the GM-CSF with IL-15 or IL-4 and TGF-β_1_ signaling pathways (37–40). Therefore, *L. crispatus* might be recognized by TLR2 and TLR6 in THP-1 cells and then activate the related TFs of the GM-CSF with IL-15 or IL-4 and TGF-β_1_ signaling pathways, which can ultimately lead to CD207 expression (seen in Figure 8C). Subsequently, the protein expression levels of the four TFs were examined by WB. The results showed that PU.1 and POLR2A were upregulated, but TAL1 and TIF1γ were downregulated, which suggested that the GM-CSF with IL-15 or IL-4 and TGF-β_1_ signaling pathways might be induced, leading to CD207 upregulation in THP-1 cells. GM-CSF with IL-15 signaling pathway skewed monocyte differentiation into Langerhans cell like cells without Birbeck granules (45). Since THP-1 cells lack detectable Birbeck granules in the presence of *L. crispatus*, it was ultimately speculated that the IL-15 signaling pathway might exert a pivotal role in promoting CD207 expression in THP-1 cells under *L. crispatus* stimulation. People have isolated Langerhans cells which lack of Birbeck granules while the cells have usual morphologic characteristics, and were CD1a, human leukocyte antigen (HLA) class II positive and normal antigen-presenting capacity (46, 47). According to the morphology, Langerhans cells have been classified into two types (48, 49). Type 1 Langerhans cells are pyramidal in shape, locate in the suprabasal layer and contains numerous Birbeck granules. Type 2 Langerhans cells are spherical in shape, locate in the basal layer with fewer Birbeck granules and shorter dendritic processes (48, 49). However, the difference of functions between the two kinds of Langerhans cells is still unknown.

In conclusion, this study reveals three important points: (1) *L. crispatus* naturally isolated from the vaginal tract induced differentiation of monocytic precursors toward LCs morphologically and phenotypically *in vitro*; (2) PGN isolated from *L. crispatus*, not the soluble products, was a key player contributing to Langerhans-like cell differentiation. PGN, with the help of SLP, can further facilitate this process. (3) PGN of *L. crispatus* facilitates CD207 expression probably by activating the GM-CSF and IL-15 signaling pathway followed by TLR2 and TLR6 recognition in THP-1 cells. These findings not only offer an appealing option for research and development of a mucosal HIV-1 vaccine by using *L. crispatus* as a mucosal delivery vehicle or PGN from *L. crispatus* as an immunologic adjuvant but also provide a theoretical basis for further investigation of the occurrence, development and differentiation of LCs from monocytes in the FGT.

## Ethics statement

The present study was approved by the Ethical Committee of the Second People's Hospital of Yunnan Province. All volunteers provided written consent and were informed of the purposes of the study.

## Author contributions

HZ and NZ: conceptualization; JS, FL, NZ, and YG: methodology; JS and FL: formal analysis and writing of the original draft; HZ and JS: funding acquisition; All authors: review and editing.

### Conflict of interest statement

The authors declare that the research was conducted in the absence of any commercial or financial relationships that could be construed as a potential conflict of interest.

## Supplementary material

Figure S1 CD80 expression of THP-1 after stimulation with *L. crispatus* THP-1 was incubated with live *L. crispatus* (ratio = 1:0–1:500) for the indicated times (0–96 h). The percent of positive cells is presented according to MFI (mean fluorescence intensity) compared to CD80 and corresponding isotype controls.

Figure S2 TEM images of THP-1 treated with *L. crispatus* and *L. fermentum*. No Birbeck granules were observed in THP-1 cells stimulated with *L. crispatus* and *L. fermentum*.

Table S1 Identification of vaginal lactobacilli uses different methods. A 16s rDNA clone, the API 50 CH System and Bergey's Manual of Systematic Bacteriology were used to identify the vaginal lactobacilli.
