# Supplementary figures and images for: Vaginal Lactobacilli Induce Differentiation of Monocytic Precursors Toward Langerhans-like Cells: in Vitro Evidence
Source: Front Immunol. 2018 Oct 23;9:2437. doi: 10.3389/fimmu.2018.02437 (PMC6211368; doi:10.3389/fimmu.2018.02437)

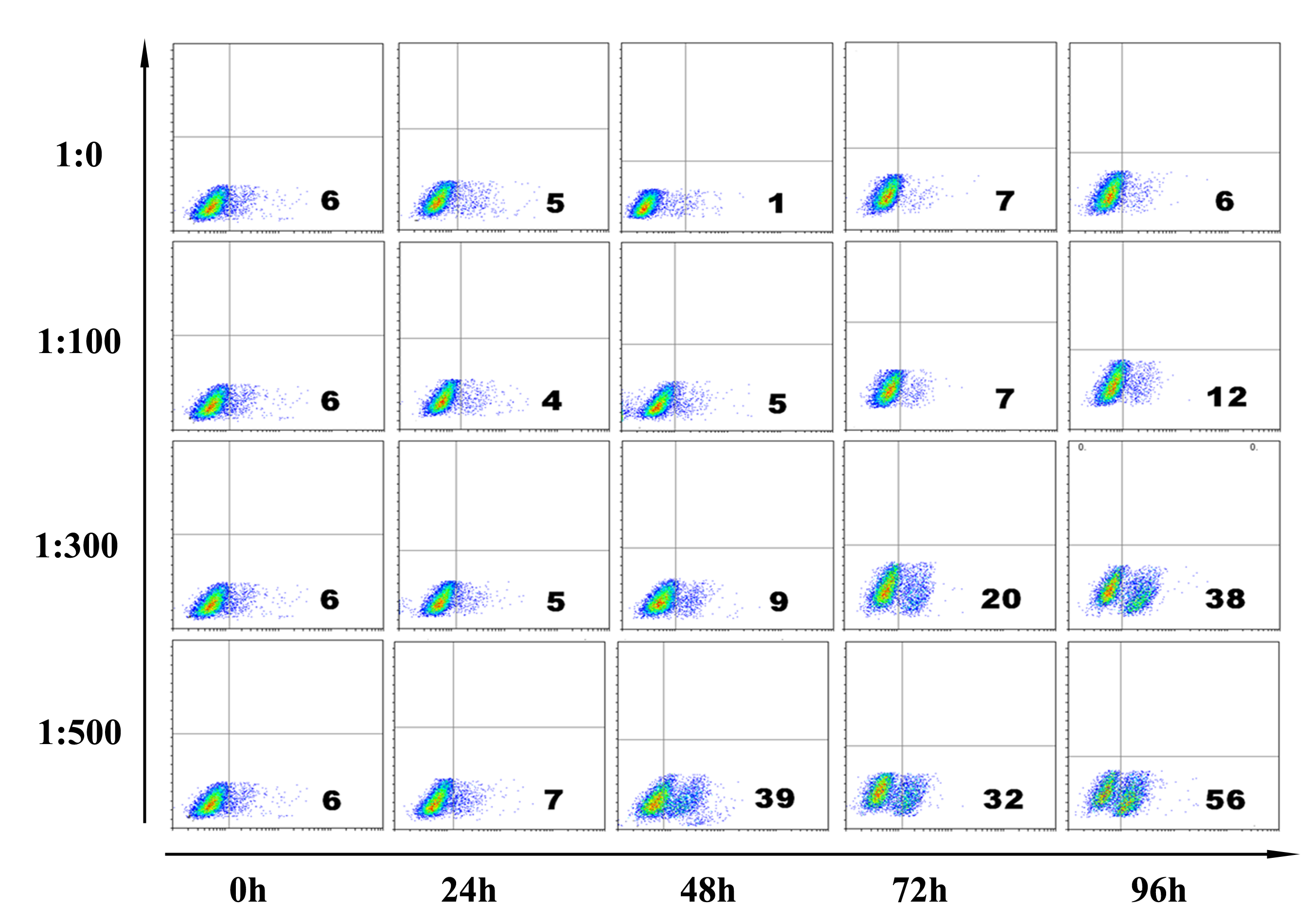

Supplement: Figure S1 — CD80 expression of THP-1 after stimulation with L. crispatus THP-1 was incubated with live L. crispatus (ratio = 1:0–1:500) for the indicated times (0–96 h). The percent of positive cells is presented according to MFI (mean fluorescence intensity) compared to CD80 and corresponding isotype controls. [file Image_1.TIF]

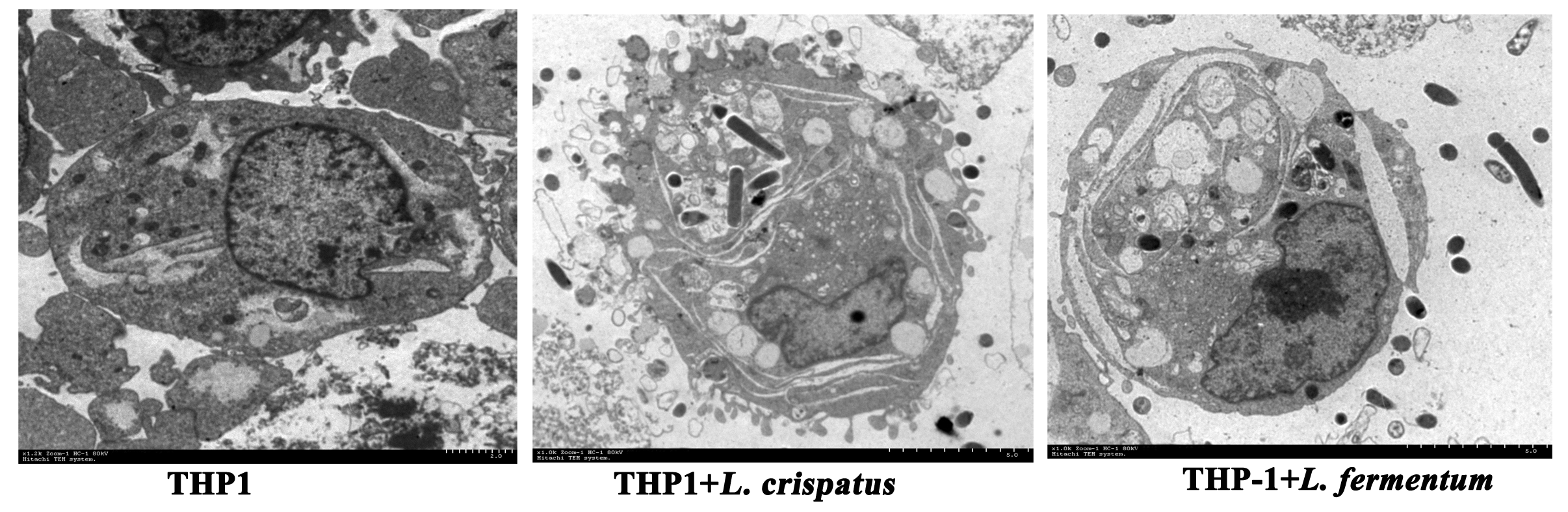

Supplement: Figure S2 — TEM images of THP-1 treated with L. crispatus and L. fermentum. No Birbeck granules were observed in THP-1 cells stimulated with L. crispatus and L. fermentum. [file Image_2.TIF]
