# Supplementary material for: Vaginal Lactobacilli Induce Differentiation of Monocytic Precursors Toward Langerhans-like Cells: in Vitro Evidence
Source: Front Immunol. 2018 Oct 23;9:2437. doi: 10.3389/fimmu.2018.02437 (PMC6211368; doi:10.3389/fimmu.2018.02437)
Supplement: Table S1 — Identification of vaginal lactobacilli uses different methods. A 16s rDNA clone, the API 50 CH System and Bergey's Manual of Systematic Bacteriology were used to identify the vaginal lactobacilli. [file Table_1.DOCX]

**Table S1. Identification of vaginal lactobacilli using different methods**

| **ID** | **16s rDNA Clone** | | **API 50 CH System** | | **Bergey's manual of systematic bacteriology** | |
| --- | --- | --- | --- | --- | --- | --- |
|  | **Name** | **Validity** | **Name** | **Validity** | **Name** | **Validity** |
| **19** | *L. fermentum* | 100％ | *L. fermentum* | 100％ | *L. fermentum* | 100％ |
| **44** | *L. jensenii* | 100％ | *L. acidophilus* | 49％ | *L. jensenii* | 100％ |
| **68** | *L. gasseri* | 99％ | *L. acidophilus* | 57％ | *L. gasseri* | 99％ |
| **15-2** | *L. delbrueckii* | 100％ | *L. delbrueckii* | 96％ | *L. delbrueckii* | 100％ |
| **17-2** | *L. crispatus* | 100％ | *L. crispatus* | 99％ | *L. crispatus* | 100％ |
| **31** | *L. johnsonii* | 98％ | *L. acidophilus* | 74％ | ND | ND |
